# Rats can distinguish (and generalize) among two white wine varieties

**DOI:** 10.1007/s10071-025-01937-2

**Published:** 2025-02-21

**Authors:** Elisa Frasnelli, Benedict D. Chivers, Barry C. Smith, W. Tecumseh Fitch

**Affiliations:** 1 CIMeC Center for Mind/Brain Sciences, University of Trento, Piazza della Manifattura 1, 38068 Rovereto, Italy; 2 School of Life Science, University of Lincoln, Lincoln, LN6 7DL UK; 3 Institute of Philosophy, Centre for the Study of the Senses, School of Advanced Study, University of London, Senate House, Malet Street, London, WC1E 7HU UK; 4 Department of Behavioral and Cognitive Biology, University of Vienna, Vienna, Austria

**Keywords:** Rat, Olfaction, Wine, Discrimination, Generalization, Categorization

## Abstract

In the olfactory literature there is considerable debate about how differences in olfactory receptors across different species map onto variations in perceptual acuity and performance. Although humans have fewer functional olfactory receptors than most other mammals, it has been suggested that linguistic and cognitive abilities help compensate for this apparent deficit and enhance discriminative abilities, particularly through humans’ ability to categorize sensory stimuli into conceptual categories. However, previous research suggests that non-human animals can learn complex categories, involving multiple perceptual dimensions, indicating that they can discriminate complex odor stimuli without language. We investigated generalization over complex olfactory categories by examining rats' discrimination of wine varieties, a challenging task for humans that has been suggested to rely heavily on human-specific linguistic, cognitive and categorization abilities. Nine rats were trained in an olfactory discrimination task (go/no-go) using a specific wine variety (Riesling or Sauvignon Blanc from different winemakers) as the S + . Rats were then tested using novel wines of the same varieties in unrewarded probe trials to assess their abilities to correctly assign instances of wine to specific categories. Interestingly, all nine rats successfully learned to discriminate the two varieties, and most rats generalized within two test trials to novel wines of the same varieties. We explore the implications of our results for olfactory concept formation and categorization more generally.

**Supplementary Information:**

The online version contains supplementary material available at 10.1007/s10071-025-01937-2.

## Introduction

Among vertebrates, mammals are considered to be olfactory specialists. For example, the number of olfactory receptor genes found in most terrestrial mammals is much larger than that seen in either fish or other tetrapods (Niimura 2009). However, there is a great deal of variability in the sense of smell within mammals, with some groups (such as cetaceans) showing extreme reductions in the entire olfactory system, and others (like primates including humans) showing relatively small numbers of viable olfactory receptor types (Niimura and Nei 2007). For example, rats appear to have roughly 1200 functional olfactory receptor genes (ORGs), while humans and chimpanzees have only 400 or so (Lledo et al. 2005; Niimura and Nei 2007). Most of this difference is probably a result of relatively recent loss of previously functional ORGs, a process termed “pseudogenization” (Gilad et al. 2004; Lledo et al. 2005; Niimura and Nei 2007), suggesting that primates have been under relaxed selection for olfactory genes. While the adaptive reasons for the loss of most olfaction in aquatic mammals may seem obvious (Niimura 2009), the reasons for a reduction among primates are less clear (Laska et al. 2000; Niimura et al. 2018). It has been hypothesized that the well-documented reduction in functional ORGs in primates reflects an increase in the importance of vision relative to olfaction (Gilad et al. 2004; Niimura et al. 2018; Niimura and Nei 2007).

However, it has also been suggested that simple counts of ORGs underestimate the human sense of smell (Laska et al. 2005; Laska et al. 2000). The few behavioral experiments directly comparing human olfactory discrimination with that of other mammals, such as dogs and rats, have documented few differences between species, at least with simple molecules and single-molecule odorants and, in some cases, humans even outperform other species such as rats (Laska et al. 2000; Passe and Walker 1985). It is possible that humans have compensated for losses at the sensory periphery both with anatomical changes (e.g., reduced air filtration in the nose, enhanced retronasal olfaction) and neural changes in central processing (Shepherd 2004). Thus, in complex tasks such as wine tasting, increased cognitive resources, including the use of language to aid olfactory memory and categorization, have been proposed to boost human olfaction beyond the simple discriminatory abilities seen in other mammals (Shepherd 2004). Thus, although the basic olfactory discrimination abilities of “normal” macrosmatic mammals such as dogs and rats are clearly excellent (Passe and Walker 1985), it remains possible that these human cognitive advantages over other mammals lead to qualitative species differences in the way olfactory stimuli are processed, categorized, and remembered. This possibility is suggested, for instance, by classical accounts of concepts according to which language is a necessary condition for conceptualization and therefore that non-linguistic species cannot have conceptual abilities (for a review see Margolis and Laurence 2023). However, considerable research on non-human animals provides support for categories being learned without language for different perceptual dimensions (visual, e.g., Nakagawa 2000, odor, e.g., Lu et al. 1993, auditory, e.g., Murphy, Mondragon and Murphy 2008; de la Mora and Toro 2014). This poses the question to what extent nonhuman animals can discriminate complex odor stimuli that involve many different perceptual dimensions.

In the experiment described here, we report a preliminary investigation of this issue by using an olfactory task widely seen as challenging even for humans: the determination of specific grape varieties in wine. Wines are highly complex chemical mixtures (Margalit 2004), and most of the sensory experience of wine “tasting” in fact derives from olfaction, with retronasal olfaction thought to play a particularly important role in humans (Smith 2019). Furthermore, any given grape variety (for example, Pinot Noir, Cabernet Sauvignon, Chardonnay, Riesling, or many others (cf. Robinson, Harding, and Vouillamoz 2013)) will vary considerably in its molecular profile depending on such issues as temperature, ripeness, soil composition and wine-making methods (Margalit 2004; Waterhouse et al. 2016). Because the task of categorizing wine variety is fundamental in wine tasting, an ability to do so is considered a pre-requisite for wine professionals, but may require considerable training. Wine variety identification is not an ability highly developed by default in ordinary untrained wine drinkers, or the population in general, but is considered a skill requiring training, exhibited by sommeliers and other wine professionals in tasting competitions (Croijmans et al. 2020). Such experts have highly developed memory skills and use a specialized wine vocabulary, suggesting that wine variety identification could present a challenge for non-verbal animals.

Here, we investigate the discrimination and generalization of distinct grape varieties, a pre-requisite for advanced wine expertise, in domesticated rats (*Rattus norvegicus domestica*). Previous work shows that rats can learn to discriminate olfactory stimuli rapidly and with high accuracy (> 90% Uchida and Mainen 2003; Zariwala et al. 2013). We chose in this first investigation to use two wine grape varieties that, from the wine professional’s viewpoint, have rather distinctive and unique characteristics: Riesling and Sauvignon Blanc. These are popular white wine varieties grown around the world, and we were able to easily purchase many distinct wines of these varieties, coming from diverse wine growing regions and involving distinct winemaking styles. We hypothesized that, despite this complexity and variety, our rats would (1) be able to learn to discriminate between wines of the two varieties during training, and (2) be able to transfer this discrimination to novel wines of the same categories during testing.

## Materials and methods

**Fig. 1:**
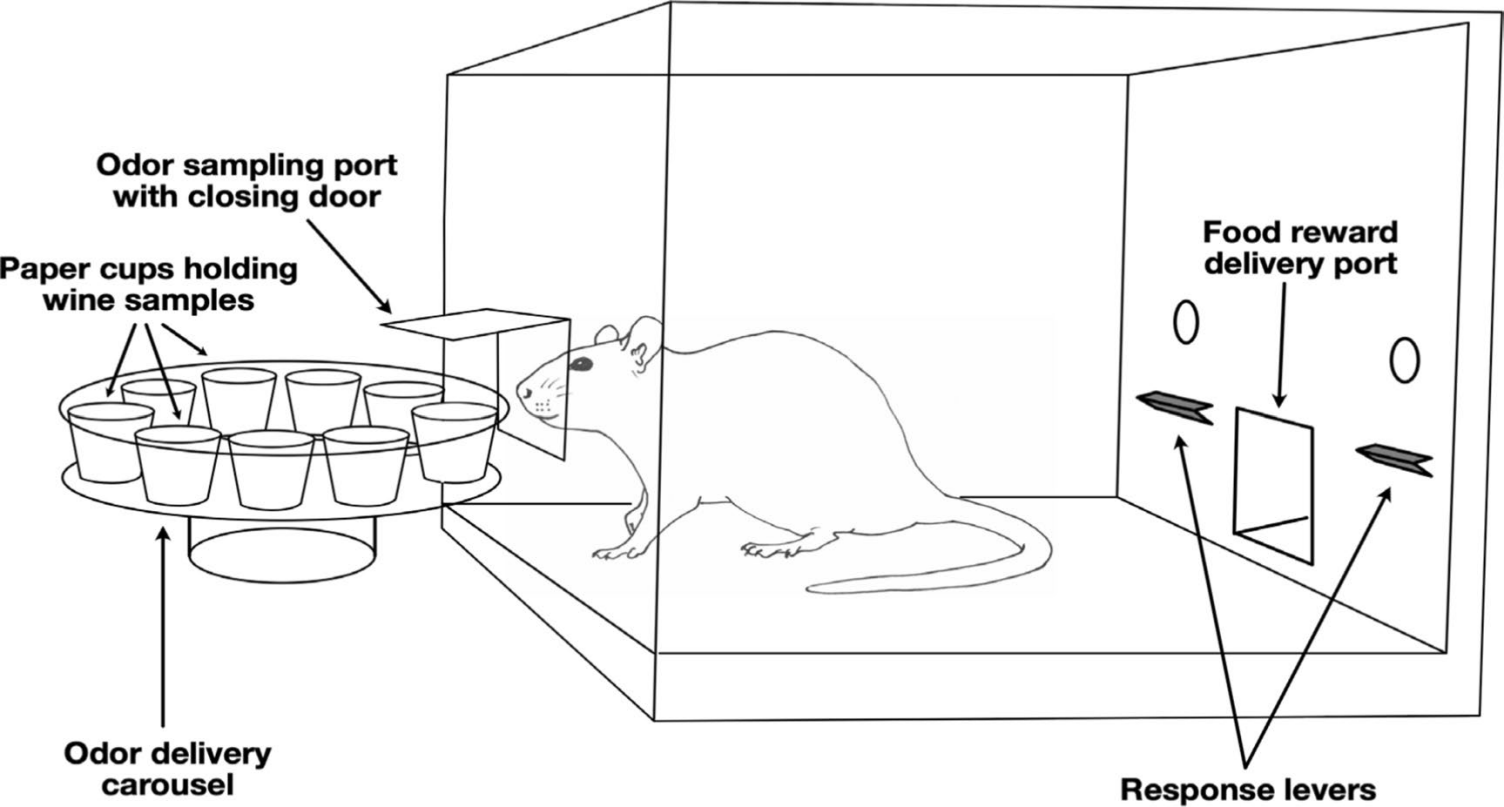
Overview of operant setup. A schematic representation of the operant chamber used in the experiment

Rats were trained to choose a class of target odorants (the rewarded S + odorants) from a set of non-reinforced odorants (S−) in an operant setup using olfactometers. Our rats had previously been trained in an experiment using simpler odors (explosives) (Keep et al. 2021), which finished 2 months before we started this experiment, and were thus already experienced in an olfactory discrimination task and our experimental setup. An overview of the system is provided in Fig. 1.

### Subjects

Uncastrated male fancy adult rats were purchased from a breeder via a pet shop. Upon arrival at the laboratory, rats (all males because the olfactory perception of female rats may change during their breeding cycle) were socially housed in groups of up to 7 individuals, and had free access to water. No animals were food- or water-deprived for this experiment. Rats were fed rodent pellets, 12 g per rat, (“Selective Rat Food”, Supreme Petfoods, Hadleigh, Suffolk) twice per day, except that on training days, and after the food-rewarded training sessions had been completed, they were fed a reduced amount at the end of the day to avoid them overeating.

### Experimental setup

Eight modular test chambers (operant boxes), model ENV-008-VP from Med Associates Inc. (www.med-associates.com) were used for this study. Each chamber was placed inside a custom-made cupboard which contained an air extraction system, consisting of a pair of plastic fans, one mounted on the left front door (intake) and one mounted on the rear wall (outtake) directly opposite. A custom-made odor delivery system was connected to each test box, consisting of a turntable, a lever arm and a carousel containing 8 odorant bays (Fig. 1, left). The carousel bays were loaded with paper cups containing filter paper bearing various odors, and the cups were covered by plastic lids to contain the odors, with a small hole facing outward (so directly under the rats’ nostrils during a nose-poke). The carousel rotated both clockwise and anticlockwise when moving between bays. For each odor presentation a bay would line up with the odor port on the operant box, the plastic lid would be lifted by the lever arm for a set amount of time (4 min in training and test sessions), and then the lid would be lowered back to its closed position. A small nose port allowed the rat to insert its nose into the odor space, where it could be detected by an infrared photobeam and detector. The system was controlled by custom programs running on the Med Associates Med-PC software on a Windows 7 PC. For further details on the setup please see previous work (Keep et al. 2021) and patent (Crome et al. 2019).

### Re-training

Rats used in this experiment had previously completed an unrelated odor categorization task using a Go/No-Go protocol previously validated on the same subjects (Keep et al. 2021) and were therefore familiar with the apparatus and procedure. Our procedure and reinforcement schedule closely followed this previous research. To refresh their familiarity, at the beginning of the present experiment, all rats were re-trained using a Go/No-Go procedure. The training odors used were a strawberry extract and a caramel extract (0.1 ml placed onto a piece of filter paper, within the paper cups in the odor delivery system; extracts from Morrison Supermarkets). Half of the rats were assigned strawberry as a rewarded stimulus (S +) and the other half were assigned caramel as S + . For the strawberry group, the caramel odor was used as the unrewarded stimulus, S−, and the strawberry odor was used as the S- in the caramel group. For each day of testing, the carousel loaded with 50% S + and 50% S−. Each training session consisted of 24 trials. Carousels held 8 odors, and thus each carousel was run through 3 times per session, with odors presented in a random order.

For each trial, an odor would be presented, a house light illuminating the chamber would light, and the rat was then allowed 20 s to perform a nose poke. Following a successful nose poke a lever would be presented (and the house light turned off), on the opposite side of the apparatus from the nose hole, and then after 5 s retracted. A correct response (lever press for an S +) led to retraction of the lever and access to a sugar pellet (preferred food type) (45 mg Dustless Precision pellets, bio-serv.com); a lever press for an S− stimulus led to lever retraction and the light above the lever being illuminated for 20 s, signaling a timeout. S + stimuli that did not yield a lever press were counted as “misses” and as incorrect. Following each lever press or retraction, an inter-trial-interval (ITI) of 20 s commenced followed by start of the next trial and presentation of the next odor. For all stages of discrimination training, the criteria for “success” were set at > 80% correct true positive (TP) responses and < 20% false positive (FP) responses, during a single training session. Individuals were given two training sessions on weekdays between the hours of 13:00 and 15:00.

### Experimental training: wine variety discrimination

Once rats successfully reached criterion on the retraining, they advanced to the grape variety discrimination. Rats were trained using a Go/No-Go procedure equivalent to that described above. Each rat was assigned, in the order they completed re-training, alternately to either the Sauvignon Blanc or Riesling group (where the group name corresponds to the S + stimulus, with odd numbered completers to Sauvignon Blanc, and evens to Riesling). This ensured a relatively even distribution of "fast" and "slow" rats across the two wine categories.

For each grape variety, four different wine types of distinct vintages, geographic origins, and makers were used as the training stimuli (Table 1). For the Sauvignon Blanc group, the S + positive odors were produced from the Sauvignon Blanc grape variety, and the S− wines from the Riesling variety. For the Riesling group, the contingency was reversed, using the same bottles. Wines were selected simply based on the grape variety from which they were made, and their availability at the time of the experiment from our main supplier, Berry Brothers & Rudd (www.bbr.com; London, UK). The only criterion was that they must be pure varietals of the two specified varieties, and we aimed to have as large (globally) a coverage as possible. For each session, the carousel was loaded with 4 S + and 4 S− odorants which were presented in a random order, but with the constraint that members of the S + or S− stimulus categories were never presented more than 3 times in a row. For each training session, the entire carousel was run through 3 times resulting in 24 trials per session (12 S + and 12 S− presentations).

**Table 1:** Training wines. Wine bottles used as experimental training odours

Variety	Name/ Source	Location	Year	ID
Sauvignon blanc	Sancerre, David Sautereau	France	2017	T1
	Pouilly Fume, Domaine Patrick Coulbois	France	2014	T2
	Sancerre Blanc, Brigitte et Daniel Chotard	France	2017	T3
	Chilean Sauvignon Blanc, Loma Larga	Chile	2015	T4
Riesling	Rheingau Riesling, Eva Fricke	Germany	2016	T5
	Von Blauem Schiefer, Heymann Lowenstein Mosel	Germany	2014	T6
	Riesling Vieilles Vignes, Domaine Lucas and Andre Rieffel	France	2016	T7
	Loibner Steinertal, Smaragd, F.X Pinchler	Austria	2012	T8

Again, each trial began with the presentation of an odor followed by illumination of the house light, and rats were required to perform a nose poke into the odor port, which turned off the house light and led to the presentation of the lever for 5 s. During a positive (S +) trial, a single lever press within 5 s led to the lever being retracted and a sugar pellet being dispensed; if a rat did not respond to the positive stimulus the lever was retracted and the program moved on to an ITI of 20 s. During negative (S−) trials, a lever press within 5 s led to the lever being retracted and a timeout; if the lever was not pressed within 5 s it was retracted and a 90 s timeout occurred, throughout which a light above the lever was illuminated. Following the timeout, after a 20 s ITI the next trial began. All rats received 2 training sessions a day. Learning criteria were again set at > 80% true positive (TP) and < 20% false positive (FP), now over 3 consecutive training sessions. Once rats reached learning criteria, they proceeded to the test session.

### Experimental testing: novel wines

The test sessions again used a Go/No-Go procedure. For each test session, the carousel was loaded with 6 of the previously learned training smells (3 S + , 3 S−), which were randomly assigned per session and positioned in random bays between 1 and 6 in the carousel. Bay 7 contained the novel S + odor and bay 8 contained the novel S− odor. These test odors were 3 novel bottles of wine of each variety (Table 2) which acted as novel S + and S− for each group respectively. For each testing session, the carousel was run through 3 times, presenting the known odors 4 times and the novel S + and S− one time in each rotation, resulting in 30 trials per session (12 S + and 12 S− known odors; 3 S + and 3S− novel odors).

**Table 2:** Testing wines. Wine bottles used as novel test odours

Variety	Name/source	Location	Year	ID
Sauvignon blanc	Sidebar, High Valley	USA	2015	1
	LuLu, Hewitson	Australia	2017	2
	Marlborough, Churton	New Zealand	2016	3
Riesling	Joseph Cattin, Alsace	France	2015	4
	Mosel, Prädikatswein	Germany	2016	5
	Gun Metal, Hewitson	Australia	2013	6

Animals received food reward for correct responses to the training S + stimuli, but no reward (nor timeout) for the novel test wines. All odors were presented in a random order and the order of the test stimuli for presentation across the test sessions was also randomized. Each animal had six test sessions and was presented with each novel S + and S− stimuli six times. The crucial data to evaluate generalization to the new wines are the lever presses to these novel S + and S− stimuli. The data from the test stimuli for each test session were only used for analysis if the rats maintained the criteria of > 80% TP and < 20% FP for the known (training) stimuli. If a rat failed to meet criteria and the data for that session were discarded, the session was repeated when the rat had completed other test sessions. The data from nine sessions were discarded for failure meet to criteria (one rat failed 3 sessions, one rat failed 2 sessions, and four rats failed 1 session).

### Stimuli

Wine samples were sourced from Berry Bros and Rudd (www.bbr.com). All wines were stored at 9 °C in an incubator away from direct sunlight. One hour before each session, ~ 5 ml of each wine was transferred to a clean receptacle and allowed to reach room temperature to standardize the odor conditions. Immediately before the sessions, 0.5 ml of wine was pipetted onto filter paper at the bottom of clean cups and covered with a plastic lid ready for placement in the carousel.

### Analysis

Statistical analyses were conducted in R 3.4.3. The key analyses sought to determine whether there were significant differences between the number of responses to the novel S + and S− odors. As a first, simple statistical evaluation we used a non-parametric Wilcoxon matched-pairs signed-ranks test to test the hypothesis that rats were more likely to press the lever for novel wines as the same variety that they were previously rewarded for (“correct” generalization) than to press for wines of the different variety (“erroneous” generalization). We used the number of “response” trials (those in which the rat pressed the lever) and treated individual rats as the independent variable (N = 9).

In a more complex analysis, which allowed us to control for individual rats and wines as random effects, we used a generalized linear mixed model with a binomial error distribution, and random effect terms for individual bottle and rat identity, to control for the varying responsiveness of each animal or to each wine.

This was implemented in R using the *glmer* function of the *lme4* package (Bates et al. 2015). The model included a binary response (with a logit link function) for “lever press/no lever press”, fixed terms for stimulus type (S + /S−) and wine variety (Sauvignon Blanc/Riesling), and random effect terms for individual bottle and rat identity. Additional interactions of individual wine bottle with other variables were also tested. Significance was determined using a likelihood ratio test comparing the full model to one lacking the fixed effect (Crawley 2015). Data are graphically presented as mean proportion of lever presses, with error bars indicating standard error.

## Results

We initiated the re-training procedure with 12 rats (6 in the caramel group, and 6 in the strawberry group). All passed criteria except for one rat belonging to the strawberry group which was then excluded from further experimental training with the wines. To reach re-training criterion, 3 rats took 4 sessions/2 days, 5 rats took 6 sessions/3 days, 1 rat 14 sessions/7 days, 1 rat 15 sessions/8 days, and 1 rat 20 sessions/10 days.

From the Sauvignon Blanc group, 5 rats (out of 6 initially) completed wine training to criterion. The only rat that did not reach criterion was the one which took 20 sessions to complete re-training. To reach criterion, each of the 5 rats in the Sauvignon Blanc group took respectively 37 (Darren), 41 (Lionel), 64 (Thomas), 65 (Peanuts) and 67 (Schroedinger) training sessions. From the Riesling group 4 rats (out of 5) completed training to criterion: the rat who failed had reached re-training criterion after 6 sessions. To reach wine training criterion, each of the 4 rats in the Riesling group took respectively 31 (Badger), 32 (Hero), 34 (Edison), and 67 (Shaun) sessions. Thus, a total of 9 rats reached training criterion, and moved on to experimental testing with the novel wines. All 9 rats then completed the experimental testing at criterion.

### Generalization data

**Fig. 2:**
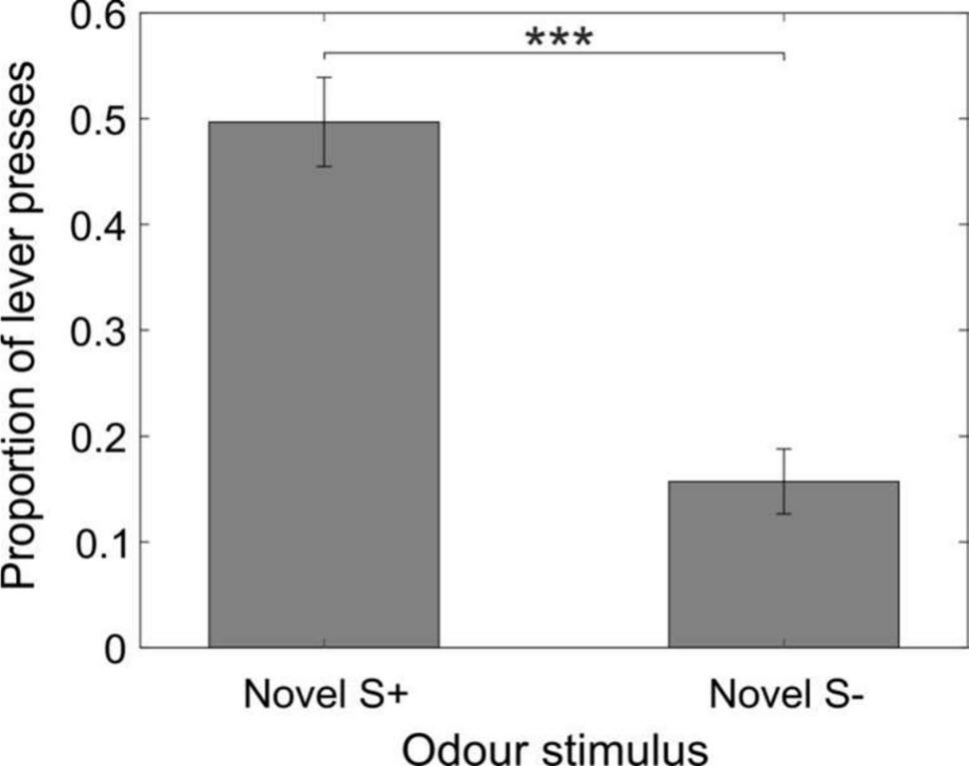
Response to novel wines. Average proportion ± SEM of indications to the novel odors from all rats (n = 9). Novel S + and S− indicate novel wines belonging, respectively, to the rewarded and unrewarded wine groups

Rats successfully generalized to the new wines: A non-parametric Wilcoxon matched-pairs signed-ranks test indicated that rats were more likely to press the lever for novel wines of the same variety that they were previously rewarded for (“correct” generalization to novel S + stimuli) than to press for wines of the different variety (“erroneous” generalization, pressing to novel S-) (‘wilcox.test’ in R with continuity correction, T = 1.5, *p* = 0.01463). This significant overall difference is illustrated in Fig. 2.

**Fig. 3:**
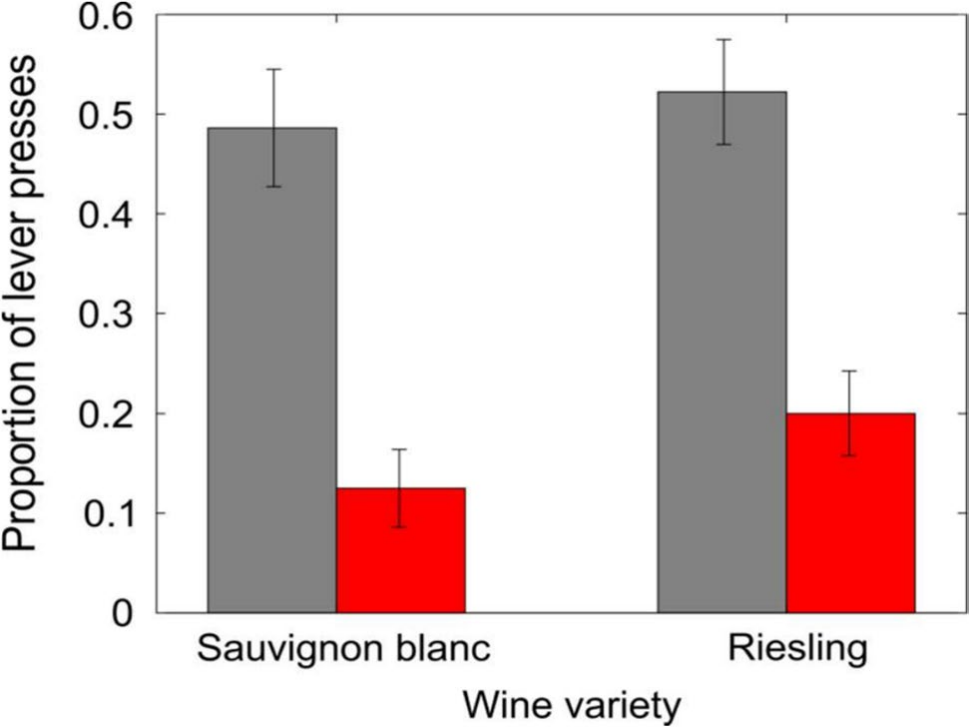
Responses to each wine type. Average proportion ± SEM of correct and incorrect indications to each of the different wine types. Grey bars: correct lever press (to trained S + variety). Red bars: incorrect lever press (to S− variety). Sauvignon Blanc; n = 5. Riesling; n = 4

In the GLMM analysis, overall, rats were significantly more likely to respond to the novel S + stimuli than to the novel S− stimuli (χ^2^(1) = 53.03, *p* < 0.001). The average proportion of responses to the novel S + stimulus was 0.5 ± 0.04, with the average proportion of responses to the novel S− being 0.16 ± 0.03 (Fig. 2). The proportion of TP and FP responses was not different between the two different wine varieties (Fig. 3).

Despite this successful generalization, all rats showed a clear generalization decrement: when comparing the novel wines with the already familiar trained wines, the overall percent correct was much lower for the novel wines. Overall percent correct for the trained wines was 94% (range for individual rats 87–97%), while for the novel wines it was only 65% (range 44–72%). This generalization decrement was highly significant (Wilcoxon matched-pairs test, T = 0.0, *p* = 0.004).

Examining only first session results, for novel wines only, rats were overall more likely to answer correctly than incorrectly (32 correct out of 54 total, or 59% correct). Given the small sample size, this difference was not statistically different from chance (binomial test, one-tailed, *p* = 0.11). However, when the first two sessions were examined (68 correct out of 108 total, or 63% correct), the generalization to the novel wines differed significantly from chance (binomial test, one-tailed number of “response” trials, *p* = 0.0045). Given that these trials received neither positive (food) nor negative (timeout) reward, this seems likely to be a sample size effect, and this rapid successful generalization seems unlikely to have resulted from any training effects. However, it remains possible that, even without reinforcement or punishment, the rats initially reacted to the novel stimuli as novel in the first testing session, but then rapidly adjusted to this novelty and assimilated them to the learned category in the following sessions.

**Fig. 4:**
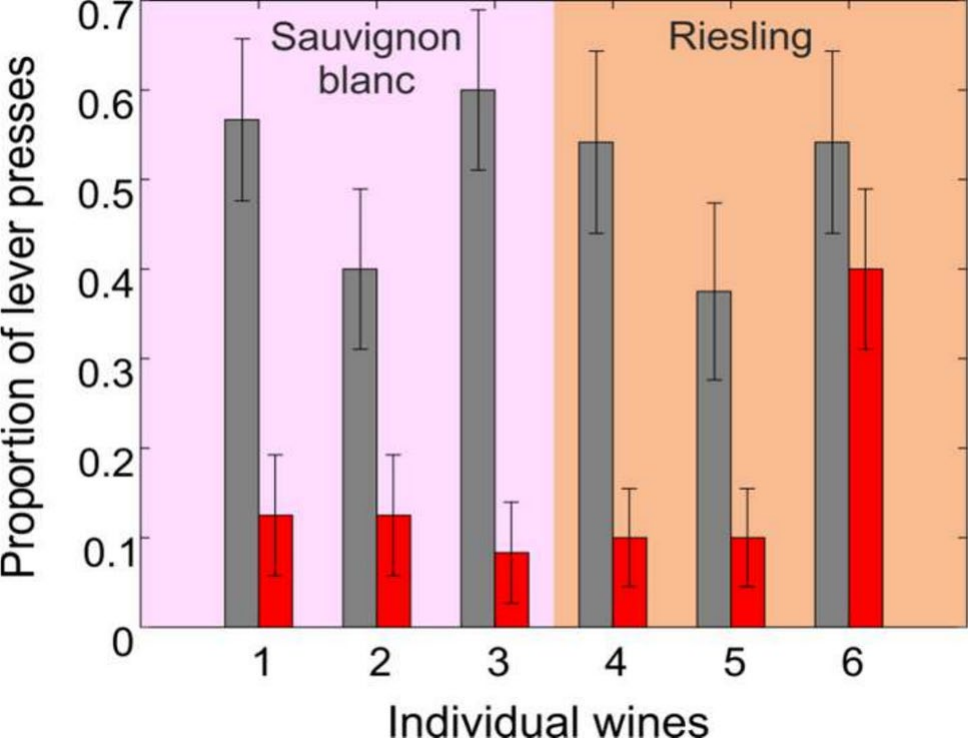
Responses to each novel wine. Average proportion ± SEM of indications to each of the novel test wine odors. See Table 2 for wine labelling. Grey bars: correct lever press. Red bars: incorrect lever press. Sauvignon Blanc; n = 5. Riesling; n = 4

The results for each individual bottle are presented in Fig. 4. As evident from Fig. 4, rats successfully generalized to each of the individual wines except for Bottle 6, the “Gun Metal” Riesling from Hewitson (see Discussion).

**Fig. 5:**
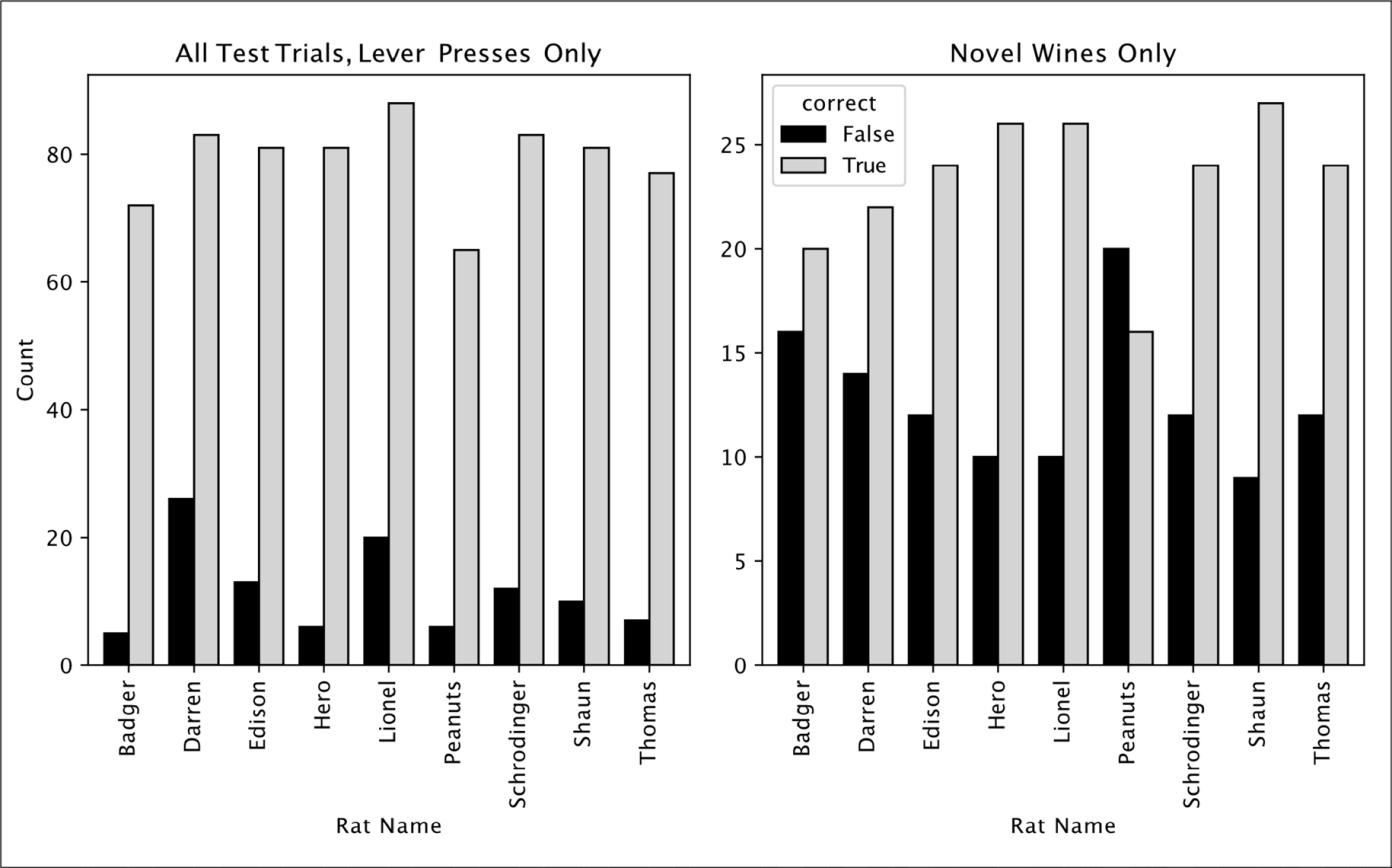
Individual responses to novel wines. Number of correct and incorrect lever presses for each individual rat, to all wines in test session (left) and to novel wines only (right). Grey bars: correct lever press. Black bars: incorrect lever press

Finally, the discrimination results for each individual rat are shown in Table 3 and Fig. 5. Eight of nine individual rats were more likely to respond positively to the novel S + than to the novel S− and six out of nine rats were significantly more likely to respond correctly to the novel wines (binomial test assuming random response rate of 50%, see Table 3). Successful generalization was thus not limited to a few “high-performing” rats, but is characteristic for most of our trained rats. However, rats adopted individually different response strategies. Some rats rarely pressed the lever to either of the novel stimuli (e.g., Badger) while others tended to press for almost all novel presentations (e.g., Lionel), but in both cases they pressed more frequently for the novel wines of their own individual S + training variety. Only one rat (Peanuts) clearly failed to generalize, giving more incorrect than correct responses to the novel wines. These individual differences in response rates to novel wines are unsurprising, since all trials involving novel stimuli were unrewarded.

**Table 3:** Individual subject’s performance

	All test trials						Novel wines only					
Rat name	F	T	N	% Correct	binom_p	sig	F	T	N	% Correct	Binom_p	Sig
Badger	23	157	180	87.2%	0.0000000	***	16	20	36	55.6%	0.3089	
Darren	33	147	180	81.7%	0.0000000	***	14	22	36	61.1%	0.1215	
Edison	21	159	180	88.3%	0.0000000	***	12	24	36	66.7%	0.0326	*
Hero	15	165	180	91.7%	0.0000000	***	10	26	36	72.2%	0.0057	**
Lionel	23	157	180	87.2%	0.0000000	***	10	26	36	72.2%	0.0057	***
Peanuts	31	149	180	82.8%	0.0000000	***	20	16	36	44.4%	0.7975	
Schrodinger	19	161	180	89.4%	0.0000000	***	12	24	36	66.7%	0.0326	*
Shaun	19	161	180	89.4%	0.0000000	***	9	27	36	75.0%	0.0020	**
Thomas	20	160	180	88.9%	0.0000000	***	12	24	36	66.7%	0.0326	*
**All**	204	1416	1620	87.4%	0.0000000	***	115	209	324	64.5%	0.0000	***

Summarizing, six out of nine of our trained rats were able to successfully discriminate novel wines of the variety rewarded during training from novel wines of the previously unrewarded variety. These results applied in general to novel varieties, with one exception to be discussed further below.

## Discussion

Our results show that rats are able, after suitable training with several exemplars of a given wine variety (Sauvignon Blanc or Riesling), to successfully generalize their recognition to new exemplars of this same variety. Because wines are very complex compounds, with many volatile compounds that vary in both presence or absence and concentration (“odor profiles”), this indicates that a nonhuman mammal can discriminate between complex odor categories. This finding is consistent with the idea that many nonhuman mammals have olfactory abilities that match or exceed those of humans, as well as genetic and neurological considerations discussed above (e.g., Lledo et al. 2005). Furthermore, it is in line with previous findings showing that matching-to-sample using olfactory stimuli can be readily performed by rats (e.g., Lu et al. 1993; Peña et al. 2006), despite their long-known difficulties with visual match-to-sample tasks (Slotnick 2001). It is also consistent with recent human data indicating that verbal naming does not play a central role in olfactory perception of wines, either in experts or novices (Croijmans et al. 2020).

From the perspective of what is known about how humans distinguish and classify wines, our results perhaps appear more surprising, and clearly support the contention that nonhuman animals (hereafter "animals") can both learn and generalize complex olfactory concepts. Our rats were trained to recognize an odor profile for either Sauvignon Blanc wines or Riesling wines, and most of them were able to project these learned odor profiles to novel wines made from Sauvignon Blanc and Riesling. Although our sample wines of each category are made from the same grape variety, they are nonetheless quite variable because they were made by different wine makers in different soils and climates in different parts of the world. This variability means, for example, that novice tasters can readily distinguish Sauvignon Blanc wines from New Zealand and France (Parr et al. 2007).

Regarding the role of language and wine “concepts”, our rats clearly had no linguistic categories to aid their discrimination performance. Our results suggest that higher-level cognitive abilities and language are not necessary to successfully discriminate and categorize complex olfactory stimuli (cf., Slotnick 2001; Shepherd 2004). Interestingly, verbalization also does not appear to be crucial in human wine tasting (Croijmans et al. 2020). Furthermore, there is clear evidence that novices and experts perform equally well in free sorting tasks (Ballester et al. 2009) and are similar in discriminatory abilities (Ballester et al. 2008; Danner et al. 2018). Thus, rats doing a purely olfactory task, as in the current study may serve as a useful model for how naïve humans sense wine aromas.

Our data contradict classical accounts of concepts, which argue that language is a necessary condition for conceptualization and that non-linguistic species therefore lack conceptual abilities. Brandom (1994), for example, has argued that representations in animals are reducible to discrimination; in our case, this would imply that rats can simply discriminate between varieties of wine (similarly to human novices), but are unable to form wine categories or “concepts.” These arguments are inconsistent both with the current results, and multiple previous studies showing that animals, both vertebrates and invertebrates, are able to discriminate, categorize and generalize visual stimuli (Herrnstein 1979, 1985; Wu et al. 2013). In pigeons, for example, differential reinforcement was shown not to affect the discriminability of individual instances of images belonging to the “tree” category (Herrnstein 1979). Furthermore, pigeons seem to use two non-mutually-exclusive mechanisms to put stimuli into the right category (Herrnstein 1985): (1) they extract abstract rules of categorization and (2) they learn to identify visual stimuli down to small details (i.e., photographic retention), and retain much of what they learned for a year and more. Our data support the case that non-human animal cognition supports some form of conceptual capabilities (e.g., Bermudez 2003; for a review, see Margolis & Laurence 2023), although the question remains open of whether substantial differences remain between human linguistically grounded concepts and other types of perceptual categories.

The high generalization performance of our rats, almost all of whom showed successful generalization to novel wines, may be partly explained by our training regime, which used a variety of target stimuli intermixed in a single training session (cf. Caldicott et al. 2023). Most previous work training animals to detect multiple odors or odor categories (mostly in the context of explosive- or drug-sniffing dogs) has used a sequential method, where an animal is trained on single target odorants until reaching criterion, and then the target odor is changed. However previous work comparing these and other methods has shown that combining multiple olfactory target stimuli in a single training session yields better olfactory generalization in both dogs and rats (Lazarowski 2019; Keep et al. 2021). Our results are consistent with previous work on pigeon visual generalization (e.g., Blough 1972) and theoretical models of generalization (Ghirlanda & Enquist 2003).

Turning to categorization, our rats were trained on two olfactory categories, counterbalanced across individuals, and most of them generalized their decisions to novel members of the same category. This is consistent with a long history of animal categorization based on visual stimuli (e.g., Herrnstein 1979, 1985). However, very few previous studies have examined olfactory categorization in animals. Johnston & Jernigan (1994) demonstrated that male hamsters can categorize distinct scents from the same familiar female from another unfamiliar female, suggesting that "individual" forms a category for these biologically relevant odors. The first study clearly demonstrating olfactory categorization for non-biologically-relevant stimuli (Wright et al. 2017) showed that dogs trained to identify various accelerants on diverse substrates could generalize their learned category to novel stimuli. This study adapted Hernnstein’s elegant “pseudo-category” method to show that this could be explained neither by rote learning nor other low-level explanations (e.g., a “feature positive” effect, Wright et al. 2017). Our study thus takes its place in a small but growing set of studies demonstrating that animals are capable of learning and generalizing arbitrary categories in the olfactory domain.

Turning now to individual differences, six rats that completed training successfully discriminated novel wines in the test phase: our results cannot be explained by one or a few high-performing individuals. However, there were individual differences in response probabilities to the novel wines, perhaps explained by the fact that no reward, positive or negative, was provided for test wines. Similarly, humans often show considerable individual differences in wine tasting tasks (e.g., Eriksson et al. 2012). The individual differences seen in our rats are perfectly consistent with these and other well-known examples of human variability, but it remains unknown whether consistent genetically based individual differences in odorant perception exist in rats.

Finally, an interesting oddity in our study was that our rats did not consistently distinguish novel wine No. 6, a Riesling, from a novel Sauvignon Blanc (Fig. 5). This outlier wine was a Hewitson Riesling made by the same wine maker from the same region in Australia as one of our contrasting wines, the Hewitson Sauvignon Blanc. One possible explanation for our rats’ lack of discrimination between grape varieties in this case is that the rats were picking up on a similarity across these wines, due to the signature of the wine maker or winery. The Hewitson Riesling, when tasted by the authors, lacked the typicality of other Rieslings from this region, and indeed showed marked similarities to the Hewitson Sauvignon Blanc. This suggests that our rats may have been picking up on objective similarities in the odor profiles due to wine-making style and/or growing conditions, obscured by the "Riesling" categorization. If this hypothesis is correct, one future line of work would be to explore rats’ ability for perceptual learning of other, less obvious categories relevant to human wine tasting, such as maker or region of origin.

## Limitations

Although the number of rats used in this study was small, the results were consistent among rats (see Fig. 5 above). This suggests that the results documented here apply to male laboratory rats in general, although further research would be needed to determine if the same applies to females. A larger concern regards the degree to which our results could be generalized to other varieties of wine as both of our wines have a rather distinctive odor profile (Parr et al. 2007), and it is likely that two more closely related grape varieties may be less discriminable.

The most significant issue left open by this work is the degree to which the odor profiles used by our rats to perform these discriminations overlaps with those used by humans. Do rats and humans form similar perceptual “odor objects” (Stevenson & Wilson 2007)? Given the complexity of odorants in any wine, it may be possible for a trained rat to use one or a few compounds to discriminate a variety, while a human wine expert may rely on many different odorants (Parr et al. 2007). However, the degree to which even individual humans use similar perceptual strategies to discriminate wine varieties, or food flavor more generally, remains highly contested (cf. Smith 2007a, 2007b, 2017).

## Future comparative work

The work reported here shows that a nonhuman animal can, with training, learn to discriminate among different wine varieties, performing a task considered difficult for humans. This work opens numerous directions for further work in comparative olfaction. One disadvantage of rats as experimental subjects for this type of work is their relatively short life span (around two years of adulthood), and given the amount of time experiments like this require, a single trained rat might at best perform two or perhaps three experiments in its lifetime. Obviously, dogs or pigs (both highly trainable and famous for their olfactory abilities) would be useful species for future research, as would elephants (which have an unusually high number of olfactory receptors, even among mammals: Niimura et al. 2014).

More broadly, we know that the number of olfactory receptor genes is highly variable across mammalian species (Gilad et al. 2004; Niimura 2009; Niimura et al. 2014), as is the size of the olfactory bulb and olfactory-related cortex (Passe and Walker 1985; Stephan et al. 1981). By comparison to the situation in color vision, where the number of functional cone opsins clearly effects perceptual discrimination capabilities (Chittka & Menzel 1992; Jacobs 1996, 2010; Osorio and Vorobyev 2008), we might expect the number of functional olfactory receptors to have strong correlates in olfactory perception. For example, given frequent claims that nonhuman primates have become “microsmatic” due to their reliance on vision, it would also be interesting to apply the approach used here to monkeys. Existing olfaction data for nonhuman primates do not support the idea that they are impaired in their olfactory discrimination and categorization (Laska et al. 2005, 2000).

A psychophysical follow-up approach would be to investigate more precisely what sorts of odor concepts are built up in nonhuman animals during olfactory training. For example, future work could use reaction times to novel wines (or novel odorants) to derive multi-dimensional scaling analyses. The current data are not well-suited to such an analysis, because rats had 20 s to make a nose poke and 5 additional seconds to press the lever; to get useful reaction times a speeded reaction time task would be more suitable. Such an analysis in nonhuman animals would allow comparisons to recent human results from free sorting tasks on wine varieties (Ballester et al. 2008). Furthermore, for direct comparison with animals, it would be ideal to have humans complete the same speeded reaction time task, and also use free sorting tasks to see their spontaneous categories (cf. Ballester et al. 2008).

Finally, it would be worthwhile to explore some more subtle differences in wine odors. Can rats distinguish red wines (e.g., the Pinot Noir grape variety characterizing Burgundy, *versus* the Cabernet Sauvignon blend characterizing Bordeaux?). Can rats or other mammals distinguish aged from new wines regardless of grape variety? Can rats distinguish the same grape variety from different locales, such as Sauvignon Blanc from different countries? Different wine regions have different climates and wine-making practices which lead to specific profiles for different countries; for example, Sauvignon Blanc varieties from France *versus* New Zealand can be distinguished by wine experts (Parr et al. 2007). Given the importance winemakers place on *terroir* (the geology, climate, soil and terrain of a wine region or vineyard) it would be fascinating to know whether non-human animals can recognize a Pinot Noir from Burgundy *versus* the same grape from the Loire or Alsace.

Thus, even the simple olfactory test set-up used here opens a host of possibilities for extensions, and an opportunity to address fundamental issues ranging from the perceptual correlates of receptor subtypes and olfactory physiology, to the objective (or subjective) nature of “taste” itself.

## Supplementary Information

Below is the link to the electronic supplementary material.

Supplementary file1 (XLSX 12 KB)

Supplementary file2 (PDF 76 KB)

## Data Availability

No datasets were generated or analysed during the current study.
