# Correction: Rats can distinguish (and generalize) among two white wine varieties

**DOI:** 10.1007/s10071-025-02005-5

**Published:** 2025-09-01

**Authors:** Elisa Frasnelli, Benedict D. Chivers, Barry C. Smith, W. Tecumseh Fitch

**Affiliations:** 1 CIMeC Center for Mind/Brain Sciences, University of Trento, Piazza della Manifattura 1, Rovereto, 38068 Italy; 2 School of Life Science, University of Lincoln, Lincoln, LN6 7DL UK; 3 Institute of Philosophy, Centre for the Study of the Senses, School of Advanced Study, University of London, Senate House, Malet Street, London, WC1E 7HU UK; 4 Department of Behavioral and Cognitive Biology, University of Vienna, Vienna, Austria

**Correction: Animal Cognition (2025) 28:16**

10.1007/s10071-025-01937-2

In the sentence beginning 'Materials and Methods' in this article, the text 'Rats were fed rodent pellets, 12 g per rat, (“Selective Rat Food”, Supreme Petfoods, Hadleigh, Suffolk) once per day' should have read 'Rats were fed rodent pellets, 12 g per rat, (“Selective Rat Food”, Supreme Petfoods, Hadleigh, Suffolk) twice per day'.

The original article has been corrected.

