# Supplementary figures and images for: Rats can distinguish (and generalize) among two white wine varieties
Source: Anim Cogn. 2025 Feb 21;28(1):16. doi: 10.1007/s10071-025-01937-2 (PMC11842533; doi:10.1007/s10071-025-01937-2)

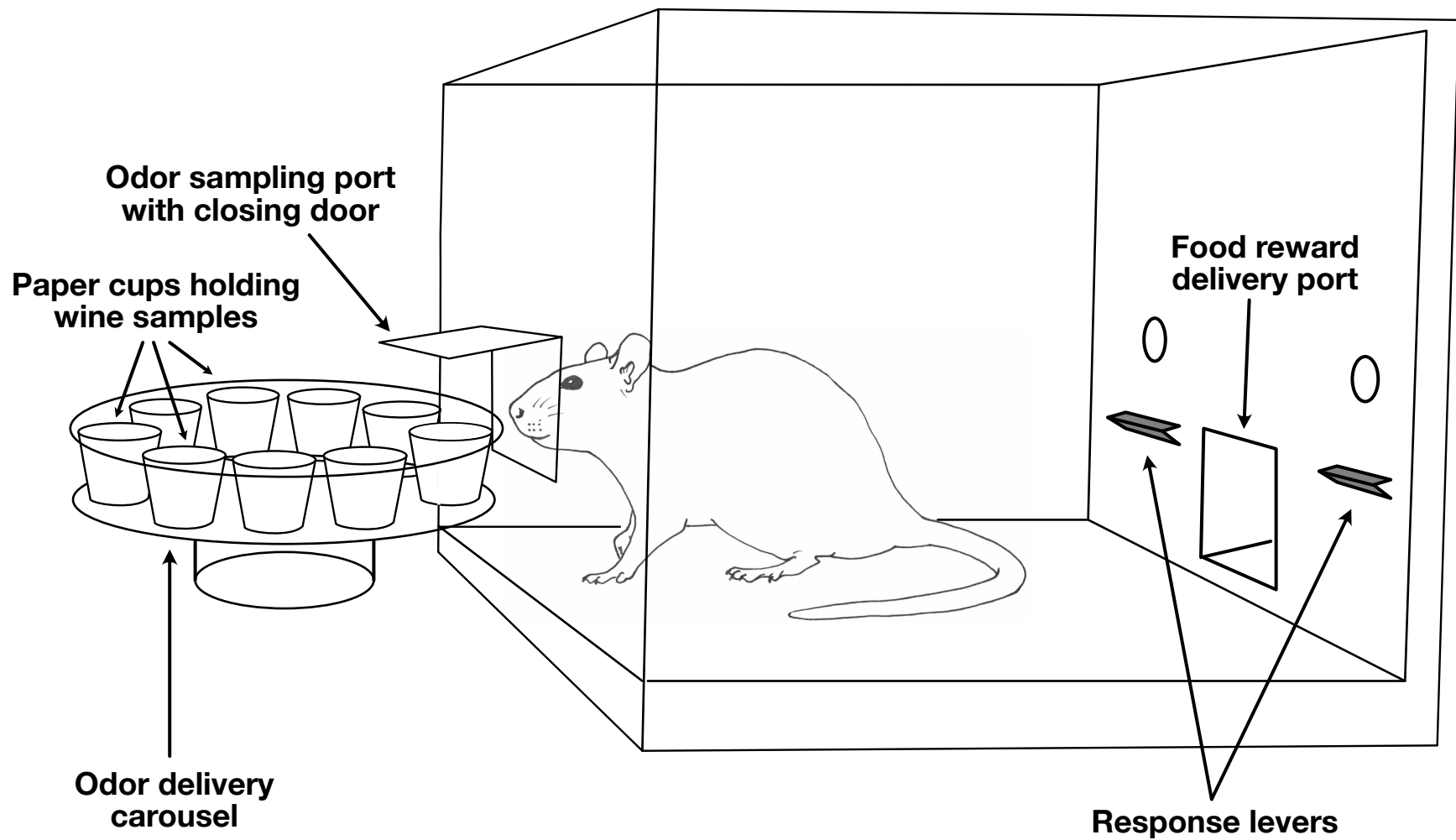

Supplement: Supplementary file 2 — Supplementary file2 (PDF 76 KB) [file 10071_2025_1937_MOESM2_ESM.pdf]
